# Dermatophytoses Caused by *Trichophyton indotineae*: The First Case Reports in Malaysia and the Global Epidemiology (2018–2025)

**DOI:** 10.3390/jof11070523

**Published:** 2025-07-15

**Authors:** Yi Xian Er, Kin Fon Leong, Henry Boon Bee Foong, Anis Amirah Abdul Halim, Jing Shun Kok, Nan Jiun Yap, Yuong Chin Tan, Sun Tee Tay, Yvonne Ai-Lian Lim

**Affiliations:** 1 Department of Parasitology, Faculty of Medicine, Universiti Malaya, Kuala Lumpur 50603, Malaysia; anishalim91@gmail.com (A.A.A.H.); kokwill00@um.edu.my (J.S.K.); nanjiunyap@um.edu.my (N.J.Y.); 2 Pediatric Institute, Kuala Lumpur General Hospital, Jalan Pahang, Kuala Lumpur 50586, Malaysia; leongkinfon@gmail.com (K.F.L.); tan_yuongchin@hotmail.com (Y.C.T.); 3 Foong Skin Specialist Clinic, Jalan Kamaruddin Isa, Taman Fair Park, Ipoh 31400, Malaysia; bbfoong@gmail.com; 4 Department of Dermatology, Faculty of Medicine, Quest International University, Jalan Raja Permaisuri Bainun, Ipoh 30250, Malaysia; 5 Department of Medical Microbiology, Faculty of Medicine, Universiti Malaya, Kuala Lumpur 50603, Malaysia; tayst@um.edu.my

**Keywords:** *Trichophyton indotineae*, dermatophytoses, itraconazole, Malaysia, antifungal, epidemiological, KOH microscopy, ITS sequencing

## Abstract

*Trichophyton indotineae* is emerging globally from its origin in India, presenting with a terbinafine resistance and causing significant clinical burden. We report herein the first four confirmed cases of *T. indotineae* dermatophytoses in Malaysia, which were diagnosed based on the microscopic examination of skin scrapings using potassium hydroxide (KOH) wet mount, followed by confirmation via culture and Internal Transcribed Spacer (ITS1) sequencing. In contrast to conventional *Trichophyton* infections, *T. indotineae* dermatophytoses demonstrate extensive cutaneous involvement and marked inflammation with erythematous lesions. All cases exhibited a chronic course lasting more than three months, with evidence of person-to-person transmission. Although one patient reported a travel to Singapore, three had no recent travel history, suggesting possible local transmission. The isolates produced characteristic white, cottony colonies with radial mycelial growth on Mycosel agar after incubation at 30 °C for four days. Three patients responded well to oral itraconazole (200 mg daily), with reduced inflammation and erythematous lesions observed two weeks after treatment initiation. The occurrence of *T. indotineae* particularly among patients without a travel history, suggests a potential endemic establishment. This fungal pathogen warrants consideration in cases of extensive or recalcitrant dermatophytoses. Further investigations into the diagnostic methods, antifungal susceptibility profiles, and epidemiological risk factors of Malaysian strains are warranted to enhance clinical management and inform public health interventions.

## 1. Introduction

Dermatophytes, comprising multiple genera including *Trichophyton*, *Epidermophyton*, and *Microsporum*, are pathogens responsible for some of the most widespread cutaneous infections globally, affecting 25% of the world’s population, particularly in tropical regions [1]. These fungal pathogens are widespread as they could be geophilic, zoophilic, or anthropophilic [2]. Dermatophyte colonization typically results in an inflammatory condition termed “tinea”, which is further classified based on the site of inflammation as tinea corporis (body), tinea cruris (groin), tinea pedis (lower limbs), or tinea unguium (nails) [3].

**Figure 1:**
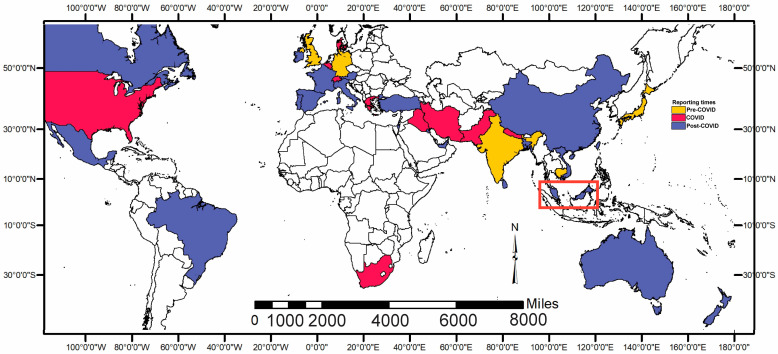
Global spread of *Trichophyton indotineae* (2018–2025). Geographical distribution showing the temporal progression of *T. indotineae* infections worldwide. Colors correspond to the chronology of reported cases: yellow indicates transmission before the COVID-19 pandemic (pre-2020), red represents transmission during the pandemic (2020–2021), and blue denotes transmission after the pandemic (2022 onwards), with Malaysia (highlighted with a red box) is among the most recent affected countries.

Among the most prevalent dermatophytes are *Trichophyton rubrum*, *T. interdigitale*, and *T. mentagrophytes* [4]. However, the predominance of specific species continuously shifts due to human and animal migration, environmental changes, and antifungal usage, especially in our globalized and industrialized era [5]. In recent times, alarming outbreaks of resistance to oral terbinafine and other antifungals have emerged worldwide. The first cases of an unusual strain of *T. interdigitale* highly resistant to terbinafine (now known as *T. indotineae*) were reported in India in 2018 [6], followed by the isolation of similar strains in other countries (Figure 1 and Table S1).

This newly defined pathogen was previously misidentified in earlier publications as terbinafine-resistant *T. mentagrophytes* or *T. interdigitale*, due to its morphological similarity to these two members of the *T. mentagrophytes* complex [6,7,8,9]. Since 2020, it has been recognized as *T. mentagrophytes* genotype VIII and formally designated as *T. indotineae* [10]. Sequencing of the internal transcribed spacer (ITS) region of the fungal 18S ribosomal DNA is currently the most accurate method for identifying *T. indotineae* [11]. The outbreak strains of *T. indotineae* predominantly harbor mutations in the squalene epoxidase gene, conferring strong resistance to terbinafine as they can continue to synthesize ergosterol despite the presence of the drug [12]. Consequently, the current pandemic of *T. indotineae* infections presents novel therapeutic challenges. Patients frequently present with extensive lesions that are inflamed and pruritic [13], often leading to social isolation due to concerns about transmission through close contact. The antifungal resistance profile of *T. indotineae* commonly results in first-line treatment failure, causing patients to endure the skin lesions for months, significantly impacting both their mental and physical health. Additionally, *T. indotineae*-infected patients frequently present with recurrent infections characterized by severe inflammation and extensive lesion distribution. Given its selective advantages, *T. indotineae* has already replaced *T. rubrum* as the dominant dermatophyte in several countries, including India [10] and China [14].

To investigate this concerning epidemiological trend, our collaborative network has initiated systematic screening protocols at participating clinical sites in Malaysia. Herein, we reported the first case of *T. indotineae* infection in Kuala Lumpur, and an additional three cases reported in the state of Perak and Kedah, at the northern region of Malaysia. All patients were local residents with minimal or no comorbidities. Unlike other *Trichophyton* species, *T. indotineae* displayed a different pattern, affecting large surface areas (>30% body surface area) of patients, with severe inflammation characterized by erythematous lesions and frequent desquamation. Persistent infections were common, as all patients had been infected for more than three months. Transmission is concerning, as similar symptoms among family members or close friends were reported by all patients. There was no travel history outside Malaysia within the past six months for three out of four cases, suggesting a possibility of local transmission. The isolates from all four cases produced white, cottony, circular colonies displaying typical dermatophyte characteristics with aerial mycelium growing radially on Mycosel agar, upon incubation at room temperature for 4 days. Two out of four patients showed poor response to standard *Trichophyton* treatments, including a patient who took oral terbinafine and the other who used topical antifungal creams (Table S3).

*T. indotineae* infection has extended its prevalence to Malaysia, leading to difficult-to-treat dermatophytoses. Its occurrence in patients without travel history indicates that the fungal pathogen may have been established locally for some time. Moreover, we also synthesize global case reports available in the current literature, integrating information on immigration and travel patterns to highlight epidemiological, clinical, and therapeutic aspects that may better inform clinicians in managing this emerging infection.

## 2. Materials and Methods

### 2.1. Ethical Approval

This research was conducted under rigorous ethical oversight and has received approval from the UMMC Medical Research Ethics Committee (MREC ID: 202439-13515, approval date: 4 April 2024). Patients were thoroughly briefed about the collection of samples and photos, as well as the analyses performed, before providing their consent. All participants gave informed consent prior to the interview as well as photo and sample collection.

### 2.2. Confirmation of T. indotineae Dermatophytoses

The diagnosis of dermatophytoses was conducted using a combination of potassium hydroxide (KOH) wet mount [15] and CLSI-M54a protocol [16]. Subjects were suspected to have *T. indotineae* dermatophytoses if they presented with unusually inflamed and extensive tinea corporis or tinea cruris lesions that persisted for months, particularly in otherwise immunocompetent individuals. Subjects were confirmed as having *T. indotineae* infections if hyphae were observed from the 20% KOH wet mount and *T. indotineae* was isolated from their samples. Briefly, skin scrapings were collected from the subjects using glass slides and enclosed in black sugar paper for transportation to our laboratory. These samples were then mixed with a few drops of 20% potassium hydroxide solution and kept at 60 °C for 15 min. The scrapings were examined under a light microscope to detect hyphal elements. Additional samples were taken from the lesions (after wiping with alcohol swabs and air-drying) and cultured on Mycosel agar (ISOLAB, Malaysia, ISO-1895) [17]. DNA was extracted from the fungal cultures using a modified boiling methods [18]. In brief, a pea-sized portion of mycelial tissue was scraped from the culture plates and transferred into a 2.0 mL microcentrifuge tube containing 500 µL of Qiagen buffer ATL (Hilden, Germany) and ten silica beads. The mixture was subjected to horizontal vortexing at maximum speed for 5 min. The lysis buffer was then discarded and replaced with 1000 µL of nuclease-free water. The tubes were briefly vortexed and centrifuged at 3000× *g* for 1 min; this washing step was repeated once to remove contaminants. Subsequently, 100 µL of nuclease-free water was added, and the tubes were incubated at 99 °C for 1 min. The lysate was immediately cooled at −20 °C for 1 min and centrifuged at 12,800× *g* for 1 min. The supernatant (lysate) was stored at −20 °C until further use. The extracted DNA was first amplified using dermatophyte-specific primers in polymerase chain reaction (PCR) assays as described by Kobylak et al., 2016 [19], followed by bidirectional sequencing of the amplified fragment for species identification, using PCR primers ITS1 (5′-TCCGTAGGTGAACCTGCGG-3′) and ITS4 (5′-TCCTCCGCTTATTGATATGC-3′), following a modified protocol based on Ferrer et al., 2001 [20].

### 2.3. Data Collections and Analysis

For each patient, a pre-tested, bilingual (English–Bahasa Melayu) interview-based questionnaire was administered. The information collected included basic demographic data, socioeconomic status, travel history, education level, lifestyle factors, pet ownership, personal hygiene practices, as well as history of medication and skin infections. Patients’ height and weight were measured and recorded. Body Mass Index (BMI) was calculated, and classified into four categories (obese, overweight, normal and underweight) based on the World Health Organization (WHO) standard [21].

In order to estimate the possible arrival times of *T. indotineae* cases into Malaysia, the data on passports issuance and foreign arrivals were retrieved from an online database set up by Malaysian government (https://data.gov.my/ms-MY, accessed on 14 April 2025). The data were further arranged and filtered according to countries and years, then exported in the format of comma-separated values (CSV) files for analysis.

To synthesize global case reports available in the current literature (2018–2025), a list of key terms (Table S2) was used to retrieve a total of 1972 publications, ranging from retrospective analysis, case reports, reviews, and research articles from PubMed using Publish or Perish v8 [22]. The results were exported as CSV files and subsequently deduplicated based on Digital Object Identifier (DOI) using the distinct() function of the dplyr package in R v1.1.4 [23]. After deduplication, the remaining articles (n = 869) underwent keyword screening, and articles reporting infections unrelated to *T. indotineae* or *T. mentagrophytes* type VIII were excluded. The remaining articles (n = 730) were further screened to remove pure review articles, animal case reports, drug efficacy studies, and similar exclusions. Finally, we filtered the remaining articles (n = 161) to include only studies that confirmed the identity of *T. indotineae* through ITS sequencing or whole-genome sequencing (WGS), resulting in a final set of 43 articles containing 171 well-documented cases (Table S1, excluding Malaysian cases).

A map was plotted using ArcMap v10.8.3 [24] to visualize the countries affected by *T. indotineae* infections and their respective years of emergence whereas the data plots were made using the ggpubr v0.6.0 [25] and the ggplot2 v3.5.1 [26] packages in R. The ITS 1 region sequences of the isolates were inspected for quality and trimmed using SnapGene v7.2.1. The resultant FASTA files were uploaded to National Center for Biotechnology Information (NCBI) Nucleotide BLAST for homology search using Basic Local Alignment Search Tool (BLAST v2.16.0). The sequence alignment of the ITS sequences was performed using MUSCLE v5.3 [27], employing the unweighted pair group method with arithmetic mean. The sequences were trimmed to an identical length (582 bp) followed by the construction of a phylogenetic tree using IQ-Tree v2.4.0 [28].

## 3. Results

There were four confirmed cases (Table S3); one case from Kuala Lumpur; two cases from Ipoh; and one case from Kedah. Three patients seemed to have transmitted the infections to their other family members as they presented similar skin lesions (Table S3). *T. indotineae* infections from these cases were clearly distinct in terms of clinical manifestations and duration of infections, as compared to the other forms of dermatophytoses. All our cases presented with extensive manifestations of dermatophytoses for up to 12 months, with multifocal lesions covering large surface areas of the body, and significantly more severe inflammatory responses than the typical dermatophytoses. With intensely erythematous lesions covering extensive anatomical regions, these infections clinically resembled eczematous or atopic dermatitis flares, in stark contrast to typical *T. rubrum* infections, which characteristically manifest as localized lesions with distinctive white to pinkish annular configurations [29].

**Figure 2:**
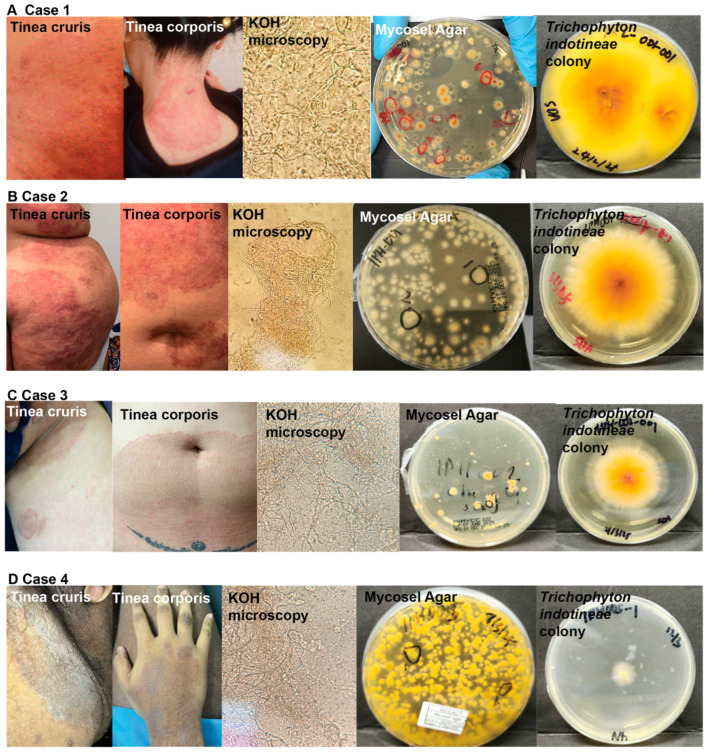
Clinical and laboratory findings from four confirmed cases of *T. indotineae* infection. (**A**–**D**) are images of tinea cruris, tinea corporis, KOH microscopy, growth on Mycosel agar, and colony morphologies of *T. indotineae* obtained from case 1–4. Representative images demonstrating the diagnostic workflow for dermatophytosis cases reported in Malaysia. (**A**) Case 1 from Kuala Lumpur showing well-demarcated, erythematous, and scaly patches affecting the nape of her neck and buttocks, KOH microscopic examination revealing fungal elements, growth on Mycosel agar, and colony morphology of *T. indotineae* isolates. Additional cases (**B**–**D**) with similar diagnostic sequence. Each case is presented sequentially with corresponding lesion photographs, microscopy, culture results, and successful isolation of *T. indotineae*.

**Figure 3:**
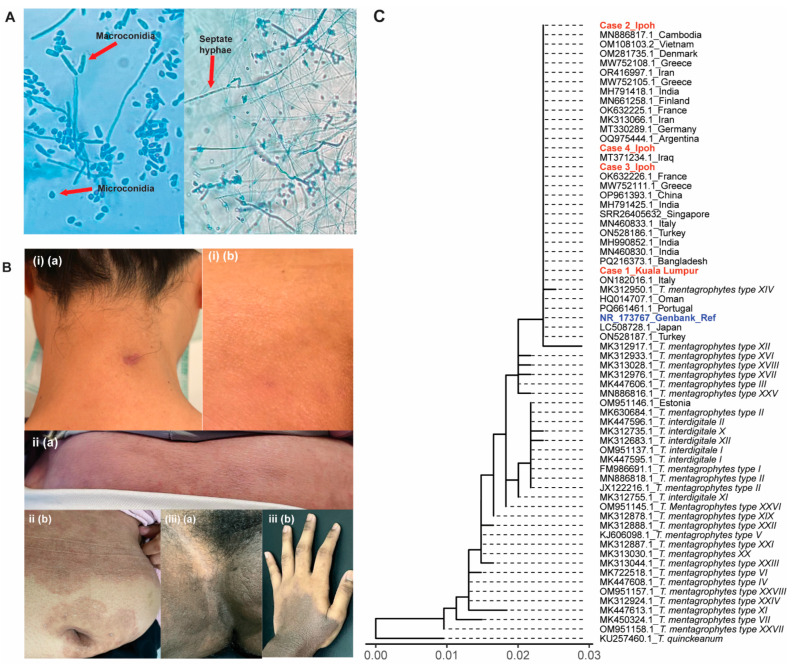
Microscopic characteristics, patients’ clinical assessment, and ITS sequence analysis of *T. indotineae*. (**A**) Lactophenol Cotton Blue (LPCB) staining: septate hyphae, club-shape multicellular macroconidia and smaller, spherical microconidia, were typical microscopic characteristics of *T. indotineae* isolates. (**B**) Clinical response of *T. indotineae* dermatophytoses after treatment. (**i**) Case 1: reduced inflammation and cutaneous lesions suggesting significant improvement were observed on the neck (**a**) and body (**b**) of the patient after 10 weeks of treatment with oral itraconazole (200 mg daily); (**ii**) Case 2: relapse of dermatophytosis on the lower back (**a**) and stomach (**b**) was suspected when the patient prematurely terminated a two-week-course of oral terbinafine prematurely (at day 7) in replacement of oral itraconazole treatment for 1 week; (**iii**) Case 4: marked inflammation was noticed on both groin (**a**) and hand (**b**) after treatment with oral itraconazole (100 mg twice daily) and topical Whitfield’s ointment for 2 weeks though hypopigmentation was observed. (**C**) Dendrogram construction from internal transcribed spacer (ITS) sequences obtained from 4 Malaysian *T. indotineae* isolates, 28 *T. mentagrophytes* (Types 1–7 and Type 10–28), and 6 *T. interdigitale* (Type 1, 2, 10–12) strains. *T. indotineae* type strain CBS 146623 (Accession: NR_173767) was used as the reference strain, while *T. quickeanum* (Accession: KU257460) was used as the outgroup [14,30,31,32,33,34,35,36,37,38,39,40,41,42,43,44,45,46].

Colonies of *T. indotineae* on Mycosel agar displayed filamentous morphology with radial growth patterns characteristic of typical dermatophyte. All fungal colonies (Figure 2) exhibited a powdery to velvety texture with pronounced yellow–orange to rust-colored pigmentation, and distinctive darker central regions but lighter, more diffuse peripheries. The concentric rings are an indication of different growth phases as the fungus expands outward from the initial inoculation point. All isolates displayed similar morphology upon staining using Lactophenol Cotton Blue (LPCB), featuring septate hyphae, club-shape multicellular macroconidia and smaller, and spherical microconidia (Figure 3A).

### 3.1. Case 1

A 33-year-old female healthcare professional of Chinese ethnicity presented with a 3-month history of diffuse pruritic dermatosis. The patient, who was otherwise immunocompetent with no significant medical history, reported international travel to Singapore several weeks prior to symptom onset. Notably, the patient disclosed that her spouse exhibited similar cutaneous manifestations, suggesting potential interpersonal transmission. Clinical examination revealed well-demarcated, erythematous, desquamative lesions distributed across multiple anatomical sites including the cervical region, facial area, abdominal wall, and gluteal surfaces (Figure 2A). The lesions exhibited atypical morphological characteristics, with coalescence forming irregular patterns, reduced border definition, and post-inflammatory hyperpigmentation—features that deviated from classical dermatophytosis. The KOH microscopy revealed the presence of hyphal elements while cutaneous scrapings were obtained for mycological culture. The culture was then subjected to molecular identification using ITS region sequencing, which definitively identified the etiological agent as *T. indotineae*, with 100% homology and 94% query coverage (555/590 bp) with *T. indotineae* type strain, CBS 146623 (Accession: NR_173767). The therapeutic management included oral itraconazole (200 mg daily) for a 3-month duration. This intervention resulted in complete clinical resolution with excellent tolerability. Significant improvement in lesion appearance and symptom reduction was documented after 10 weeks of treatment (Figure 3B(i)). A second follow-up call with the patient on day 187 (18 June 2025) confirmed complete recovery from the infection since March 2025.

### 3.2. Case 2

A 51-year-old female patient of Malay ethnicity presented to one of the authors’ clinic with persistent, pruritic, erythematous-desquamative lesions distributed across the upper extremities, superior abdominal region, and gluteal areas (Figure 2B). The patient reported symptom onset in mid-2024. Professional history revealed employment as an educator with no recent travel history. Clinical evaluation indicated class I obesity (BMI: 33.7 kg/m^2^). Prior to consultation at our facility, the patient had sought treatment at local clinics, where she was prescribed topical preparations of unlabeled composition and oral terbinafine. Following an insufficient therapeutic response, the patient was referred to our dermatological service. During a clinical interview, the patient reported that her son had similar cutaneous manifestations, suggesting potential intrafamilial transmission. Diagnostic mycological examination was performed using skin scrapings collected from the affected regions. ITS region sequencing confirmed *T. indotineae* as the causative pathogen with 100% homology and 93% query coverage (549/590 bp) with the *T. indotineae* type strain, CBS 146623 (Accession: NR_173767). Based on clinical presentation and molecular identification results, the patient was initiated on a therapeutic regimen consisting of oral itraconazole 100 mg twice daily, and topical miconazole cream bd. Follow-up assessment after 2 weeks of treatment revealed significant improvement in lesion appearance and symptom reduction (Figure S1B). However, an incident occurred when the patient failed to show up for follow-up. She chose to visit another clinic and was prescribed oral terbinafine. Subsequently, she noticed a relapse happened (Figure 3B—Case 2). A follow-up call with the patient on day 112 (18 June 2025) confirmed complete clinical recovery, with no evidence of relapse following a four-week course of itraconazole administered from mid-March to mid-April.

### 3.3. Case 3

The patient was a 47-year-old male of Chinese ethnicity residing in Ipoh with no significant medical history. He presented with extensive pruritic dermatological manifestations which began approximately 6 months prior to consultation. The patient was unable to identify a potential source of infection, reporting no recent travel history, no animal exposure, and limited interpersonal contact due to the remote nature of his online business operations. The patient initially self-medicated with antifungal cream (miconazole) followed by Elomet cream; however, due to symptom persistence without spontaneous resolution over a six-month period, he subsequently sought evaluation at one of the authors’ clinical practices in Ipoh. Physical examination revealed widespread, light-brown, desquamative, polycyclic plaques predominantly distributed across the superior abdominal region and inguinal areas (Figure 2C). Microscopic examination of cutaneous scrapings confirmed the diagnosis of dermatophytosis. Based on clinical presentation and mycological culture findings, the patient was diagnosed with extensive tinea corporis and tinea cruris. He was initiated on a therapeutic regimen consisting of oral itraconazole (100 mg twice daily) and topical Whitfield’s ointment for a duration of two weeks. Subsequent molecular identification of the fungal culture using ITS region confirmed *T. indotineae* as the etiological agent, with 100% homology and 93% query coverage (549/590 bp) with the *T. indotineae* type strain, CBS 146623 (Accession: NR_173767). Clinical assessment following the 2-week treatment was not successful, as the patient did not attend the follow-up appointment.

### 3.4. Case 4

The fourth case involved a 19-year-old male student of Indian ethnicity who was enrolled at a university in Kedah state. The patient presented at one of the authors’ clinics during a visit to his hometown in Ipoh. Clinical examination revealed extensive pruritic, erythematous plaques with well-defined, elevated borders, predominantly affecting the lower anatomical regions, including the inferior abdominal wall, inguinal areas, and lower extremities (Figure 2D). The patient reported that the lesions had persisted for approximately 12 months. Prior to consultation, the patient had self-medicated with a combination therapy containing both antifungal agents and corticosteroids obtained from local primary care facilities, resulting in minimal clinical improvement. While the patient owned two canines, he reported negligible contact with the animals during the preceding year due to his academic residence in Kedah. Notably, no similar dermatological manifestations were observed among cohabiting family members. However, the patient’s father suggested he might have contracted the infection from a close friend who was enrolled in the same university program and exhibited similar clinical manifestations. KOH microscopic examination of skin scrapings demonstrated septate hyphal elements. Subsequent mycological culture and molecular identification confirmed *T. indotineae* as the etiological agent (Figure 2D), with 100% homology and 92% query coverage (543/590 bp) with the *T. indotineae* type strain, CBS 146623 (Accession: NR_173767). Further examination and interviews revealed that the patient might have had underlying atopic dermatitis. The patient was initiated on a comprehensive therapeutic regimen consisting of oral itraconazole 100 mg twice daily, oral terbinafine 250 mg daily, and topical miconazole cream twice daily. Clinical assessment revealed marked improvement in the appearance of groin lesions after 14 days of treatment; however, the patient developed drug-related post-inflammatory hyperpigmentation in his upper limbs (Figure 3B—Case 4). The second follow-up on day 102 (18 June 2024) indicated full recovery with no signs of relapse.

### 3.5. Cases Around the Globe

The collection of global reports on *T. indotineae* cases indicates possible dual sources of infection (Figure 1 and Table S1) [6,11,13,14,30,31,32,33,34,35,36,37,40,47,48,49,50,51,52,53,54,55,56,57,58,59,60,61,62,63,64,65,66,67,68,69,70,71,72,73,74,75,76,77]. Based on changes in case numbers over the years, the dermatophytosis is thought to have emerged in India between 2015 and 2017 [6]. The Indian strains appear to have spread across the world, starting with nearby countries including Nepal and Bangladesh, as well as countries with a high influx of Indian laborers, including the United Kingdom, United Arab Emirates, and South Africa. Surprisingly, *T. indotineae* was identified among hospital isolates (collected between 2017 and 2018) in Cambodia during a similar timeframe [47]. *T. indotineae* cases were reported from seven countries soon after the Indian reports, then the transmission appeared to have been temporarily halted by COVID-19 pandemic (2020–2021), with cases reported from another nine countries during the pandemic. *T. indotineae* dermatophytoses were reported from 22 countries across the globe post-COVID-19 (Figure 1, 2022 onwards), with a total of 38 countries over six continents now reporting confirmed cases.

The recorded cases around the globe (Table S1) show 88.7% of the patients had links to endemic countries—either through relatives from these regions or recent travel to endemic areas (India, Bangladesh, Nepal, and Sri Lanka). Furthermore, similar to cases in Malaysia, the majority of patients (89.9%) experienced *T. indotineae* dermatophytoses lasting more than five months, based on 119 case reports with relevant information. Global case reports also indicated that multi-focal lesions (79.1%) are common, with the groin and genitalia (63.9%) and lower extremities (38.0%) being the most frequently affected body sites. Finally, the records showed that multi-antifungal treatment was effective in 66.3% of cases. Details provided by 42 well-documented publications reveal that 54.8% of patients presented with rash, scaling, and desquamation. Other common symptoms included erythematous, annular (ring-shaped) lesions, and pruritus (itching) in 40.5% of cases. Some patients also exhibited hyperpigmentation and burning sensations. There were 87 cases explicitly documenting no response to standard antifungal therapies. Poor response to terbinafine (68.9%) was particularly prominent, while poor response to itraconazole was observed in 26.4% of the patients [30,35,57,73]. This pattern of treatment failure was perhaps the reason why 89.1% of patients reported having recalcitrant infections, as they had been suffering from these infections for more than 5 months.

The ITS sequence analysis of 4 Malaysian *T. indotineae* isolates (Figure 3C), along with those from 28 *T. mentagrophytes* (Types 1–7 and Type 10–28) and 6 *T. interdigitale* (Type 1, 2, 10–12) strains retrieved from GenBank database, reveals high homology among the global *T. indotineae* strains, suggesting limited variation in the ITS region of *T. indotineae* strains (Figure 3C). All Malaysian strains exhibited 100% sequence identity with the global strains.

## 4. Discussion

### 4.1. Cases in Malaysia: Comparative Study with the World Records

The emergence of *T. indotineae* dermatophytoses worldwide is concerning. Similar to descriptions from Italy [13], Singapore [69], and India [6], the Malaysian patients displayed a high degree of inflammation and long periods of recalcitrant infection. This observation correlates with the compiled cases (Table S1), where most patients exhibited high inflammation and severe pruritus. Furthermore, all patients showed extended colonization by *T. indotineae* for up to 12 months, indicating the strong capability of the fungal pathogen to establish persistent infection, which again correlates with publications worldwide, demonstrating *T. indotineae*’s ability to adapt to and colonize diverse populations. Moreover, three out of four cases showed signs of familial transmission (Table S3), indicating *T. indotineae*’s high transmissibility. This likely suggests its capability to produce resilient conidia that survive in urban/industrial environments for extended periods, which could adhere firmly to the host epidermis, thereby facilitating its transmission. The extensive epidermal invasion and inflammatory skin responses observed in *T. indotineae*-infected patients may be due to enhanced keratinolytic activity and other virulence factors [78]. Finally, all patients showed good clinical improvement with itraconazole treatment, which is consistent with other publications [79,80,81] (Table S1), suggesting that Malaysian strains may not have developed resistance to triazoles yet. However, the prolonged duration and dosing regimen of itraconazole—often up to 10 weeks at 100 mg twice daily—may expose patients to risks of hepatotoxicity [82] and cardiotoxicity [83]. Antifungal susceptibility testing (AFST) is crucial to determine the resistance profile of Malaysian isolates, given previously documented cases in which patients responded poorly to both itraconazole and terbinafine (Table S1).

In terms of regional epidemiology, the Malaysian *T. indotineae* cases exhibited clinical features comparable to those reported in Vietnam [32] and Singapore [69], particularly with the presentation of persistent, pruritic, erythematous, and scaly plaques affecting the groin, abdomen, buttocks, limbs, and thighs. However, the Malaysian cases displayed a broader spectrum of lesion patterns, including the involvement of the upper body regions such as the face and back (Case 1), suggesting more extensive cutaneous dissemination. Notably, three out of four Malaysian cases had no history of international travel, pointing toward emerging local transmission. In contrast, the Singaporean patient had a recent travel history to South India, consistent with an imported case, whereas the Vietnamese case had no travel link, raising the possibility of endemic presence in Vietnam. A separate case in Japan also reportedly involved a Vietnamese patient, further indicating potential transnational dissemination within Southeast Asia [74].

Systemic oral itraconazole was the mainstay treatment in all three countries. However, Malaysian patients were more frequently prescribed additional topical antifungals—including Whitfield’s ointment, ketoconazole, and miconazole—suggesting a more complex or aggressive treatment approach compared to their regional counterparts. The Vietnamese patient responded well to oral itraconazole in combination with topical sertaconazole, while the Singaporean case achieved resolution with oral itraconazole alone. These clinical observations collectively support growing concerns that *T. indotineae* may no longer be confined to imported cases, but is establishing itself locally in parts of Southeast Asia.

### 4.2. Delay Emergence of T. indotineae in Malaysia

**Figure 4:**
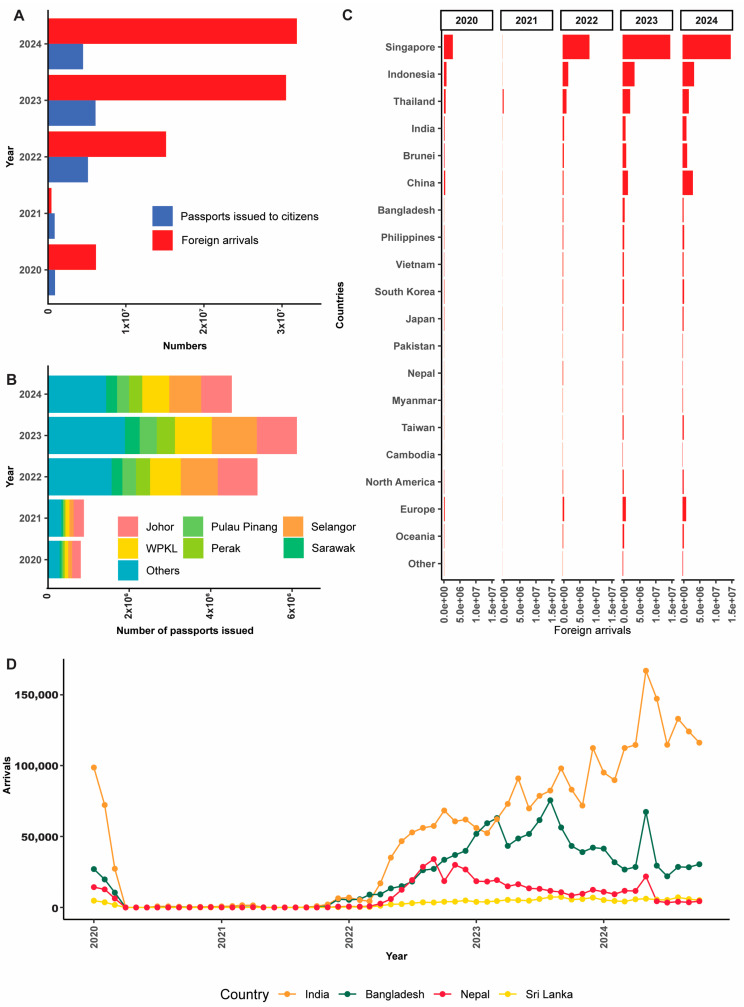
Analysis of human mobility patterns in Malaysia (2020–2024) in relation to *T. indotineae* emergence. Temporal analysis of population movement showing the following: (**A**) Annual comparison of Malaysian passports issued (blue) versus foreign arrivals (red) demonstrating restricted mobility during 2020–2021, coinciding with COVID-19 pandemic-related travel restrictions. (**B**) Geographic distribution of Malaysian passport issuance by state, highlighting interstate differences in mobility. (**C**) Foreign arrivals to Malaysia by country of origin, showing gradual recovery of international travel from neighboring regions. (**D**) Number of arrivals from the endemic countries. The detection of local *T. indotineae* cases in 2025, two years after resumption of international mobility patterns, suggests a delayed introduction of the pathogen compared to other regions or potential delayed reporting of the cases in Malaysia.

The resumption of human migration and tourism post-COVID-19 (Figure 4A,B) has led to the further transmission of *T. indotineae* infections worldwide, as most countries reported cases after 2021 (Figure 1 and Table S1). Malaysia has a high number of foreign arrivals from *T. indotineae*-affected areas (Figure 4C). Hence, it is surprising that *T. indotineae* was only detected in Malaysia in 2024. It is plausible that stricter border controls during the early post-COVID-19 period, along with a freeze on foreign worker recruitment [84], may have contributed to a delay in its introduction. These factors—combined with the lack of a formal surveillance system, limited diagnostic capacity, and widespread topical steroid misuse—likely contributed to the delayed recognition of *T. indotineae* infections in Malaysia.

Our data suggest that both the prevalence and the arrival of *T. indotineae* dermatophytoses in Malaysia may be underestimated. It is unlikely that these infections only reached Malaysia in late-2024. There are at least 2.2 million documented and up to 5.5 million undocumented migrant workers in Malaysia, with a large portion of them originating from endemic areas including Bangladesh, Nepal, and India [85]. It is highly plausible that the strains in Malaysia originated from India as we had considerable portions of the immigrant workers from India, Bangladesh and Pakistan (Figure 4C,D). There was no formal surveillance program targeting this emerging pathogen among the workers from the endemic areas according to the Foreign Medical Examination Monitoring Agency (FOMEMA), hence they could enter the countries as long as they passed the four major tests: General Physical Examination (vitals, fitness, and overall health), Blood Tests (Human Immunodeficiency Virus (HIV), hepatitis, filariasis, etc.), Chest X-Rays (tuberculosis), and Urine Tests [86]. In addition to that, two of our authors, who are dermatologists, started to encounter patients with recalcitrant dermatophytosis in 2018–2019 (Figure S1B) and most of these patients were not immigrant workers from endemic areas but local Malaysians. The local establishment of *T. indotineae* was further verified when we identified an additional 14 positive cases among locals between March 23rd and May 8th, following the discovery of the initial 4 cases. However, these cases were not included in this report due to the lack of extended follow-up information, as some patients had only just initiated treatment upon consultation. There is an urgency to alert the clinical and scientific community about the occurrence of recalcitrant *T. indotineae* infections in Malaysia. All four confirmed cases in our study were local patients, and all of them have no history of travel to India subcontinent/south Asia. Two of the patients—one who works on an online platform (Case 3) and another studying in Sungai Petani, Kedah (Case 4)—reported minimal travel activity, yet they still contracted the infections. This shows that this infection is under-reported.

The recent emergence of *T. indotineae* dermatophytoses in Malaysia may be attributed to an interwoven network of underdiagnosis and misdiagnosis, self-medication, corticosteroid misuse, and emerging antifungal resistance—factors that collectively mirror the underlying causes of the global dissemination of *T. indotineae*. Foremost among these, accurate identification of *T. indotineae* requires ITS sequencing [87], a diagnostic approach not routinely employed in most clinical laboratories, including those in Malaysia. Earlier large-scale epidemiological studies in Malaysia [88,89,90], which relied on phenotypic methods, frequently reported *T. mentagrophytes* as one of the most prevalent dermatophytes. Given the close morphological resemblance between *T. indotineae* and *T. mentagrophytes,* and the genetic differences observed in three of our strains compared to global data, the true prevalence of *T. indotineae* remains significantly under-recognized.

Furthermore, the low mortality and generally mild symptoms of tinea infections often lead patients to self-medicate with over-the-counter (OTC) antifungal products. Such practices are frequently associated with poor adherence to treatment regimens, including premature cessation of therapy before clinical cure is achieved. This issue is compounded by the widespread availability of two-in-one or three-in-one combination creams containing corticosteroids (e.g., hydrocortisone), which may provide transient symptomatic relief while simultaneously masking clinical signs, delaying diagnosis, and promoting premature discontinuation of treatment [91,92,93]. Notably, antifungal agents such as clotrimazole, bifonazole, miconazole, and terbinafine are easily accessible without prescription in many countries, including Malaysia. Subtherapeutic exposure of fungi to these agents—especially in the presence of corticosteroids—creates a selective pressure that facilitates the emergence of resistant strains [94]. In our study, two patients (Cases 2 and 3) reported the use of topical formulations containing corticosteroids. Inappropriate prolonged use of corticosteroids, in the absence of a proper diagnosis, can constitute misuse and may exacerbate fungal infections. This finding echoes similar reports from neighboring Southeast Asian countries, where corticosteroid misuse is widespread [95], and underscores the likely underestimation of the true burden and resistance profile of *T. indotineae*-associated infections in the region.

### 4.3. Limitation of the Study

This study is limited by the analysis of isolates based solely on the ITS region. The approximately 582 bp sequences, while informative, are likely constrained by their conserved nature [96]. Additionally, AFST was not performed for these isolates; thus, resistance information was inferred solely from the clinical response to treatment. Future investigations will focus on collecting a broader set of isolates from diverse cases, documenting treatment outcomes, and including AFST results from at least 30 patients to allow for more representative and robust analysis resulting in clinically relevant conclusions. Whole-genome sequencing (WGS) is also crucial, particularly for resistant strains, to identify resistance genes via sequence alignment tools including MUSCLE [27] and BLAST. Further analyses of the WGS data using single-nucleotide polymorphism (SNP) detection tools such as ParSNP [97], Snippy [98], or Gubbins [99] will be essential to resolve phylogenetic relationships among isolates from Malaysia and the global strains.

## 5. Conclusions

The recalcitrant dermatophytoses caused by *T. indotineae* displayed a delayed emergence in Malaysia despite substantial international travel from the endemic regions, likely due to enhanced post-COVID-19 border screening measures and temporary suspension of foreign worker applications. However, the actual prevalence may be underestimated due to factors such as limitations in traditional diagnostic methods, self-medication with over-the-counter antifungal treatments, and use of combination drugs containing corticosteroids that mask the typical symptoms of dermatophytoses. It is concerning that all four confirmed cases were Malaysian locals, with three having no travel history to endemic regions and three showing signs of familial transmission, suggesting probable community spread and potentially undetected cases. To address this emerging issue, there is a need to incorporate ITS sequencing for accurate identification, especially among patients with persistent and recalcitrant infections. The current epidemiological situation and the associated risk factors of this infection in Malaysia must be assessed, as there is a clear sign of local transmission from our case studies. Furthermore, *T. indotineae* infections should be suspected in cases of extensive and/or recalcitrant dermatophytoses, especially in patients with extensive erythematous lesions. Moreover, it is crucial to raise public awareness about proper dermatological care and risks of self-medication (particularly steroid misuse). Finally, there is a need to investigate the antifungal susceptibility of the Malaysian strains toward multiple different types of antifungal drugs, notably the most commonly prescribed drugs (terbinafine, itraconazole, fluconazole, clotrimazole, ciclopirox, miconazole and griseofulvin), supplemented with whole-genome sequencing of the isolates, to identify the optimal treatments for *T. indotineae* infections as well the type of resistant strains harbored by the Malaysia strains. There is also a need for further investigation on the host and *T. indotineae* interactions, both immunologically and microbiome-wise, considering the capability of this organism in establishing persistent dermatophytoses in patients.

## Data Availability

All internal transcribed spacer 1 sequences of the isolates in Case 1–4 were deposited in Universiti Malaya Research Data Repository (https://doi.org/10.22452/RD/MY1I3S, accessed on 12 May 2025) and NCBI Genbank (submission number: ascension number: PV815909-PV815912, accessed on 23 June 2025). Immigration data on passports issued and foreign arrivals were obtained from the Malaysia government (open access): foreign arrivals: https://data.gov.my/data-catalogue/arrivals (accessed on 14 April 2025); passport issued: https://data.gov.my/data-catalogue/passports (accessed on 14 April 2025). The strains were preserved in both glycerol stocks and 1% tween 80, strain request can be made once a mutual transfer agreement has been established between the institutes. Kindly email the corresponding authors to initiate the request.

## Supplementary Materials

The following supporting information can be downloaded at https://www.mdpi.com/article/10.3390/jof11070523/s1, Table S1: Case reports of global *Trichophyton indotineae* infections according to year; Table S2: List of terms searched and their corresponding number of publications. Table S3: Key characteristics of *Trichophyton indotineae* cases in Malaysia; Figure S1: (A) flow chart displaying the collection and filtering process of the published datasets and (B) Photos of suspected cases captured from different patients in 2024 prior to the start of the investigation, including different local patients with severe lesions on the lower limbs (i, ii) and on the upper extremities and chest (iii), as well as an immigrant worker (Bangladeshi) in Kuala Lumpur with lesions on the thorax and abdomen (iv).

## Abbreviations

The following abbreviations are used in this manuscript:

PCR	Polymerase Chain Reaction
KOH	Potassium Hydroxide
ITS	Internal Transcribed Spacer
SNP	Single-Nucleotide Polymorphism
COVID-19	Coronavirus Disease 2019
FOMEMA	Foreign Workers Medical Examination Monitoring Agency
WHO	World Health Organization
CSV	Comma-Separated Values
NCBI	National Center of Biotechnology Information
BLAST	Basic Local Alignment Search Tool
LPCB	Lactophenol Cotton Blue
HIV	Human Immunodeficiency Virus
