# Supplementary figures and images for: Dermatophytoses Caused by Trichophyton indotineae: The First Case Reports in Malaysia and the Global Epidemiology (2018–2025)
Source: J Fungi (Basel). 2025 Jul 15;11(7):523. doi: 10.3390/jof11070523 (PMC12298097; doi:10.3390/jof11070523)

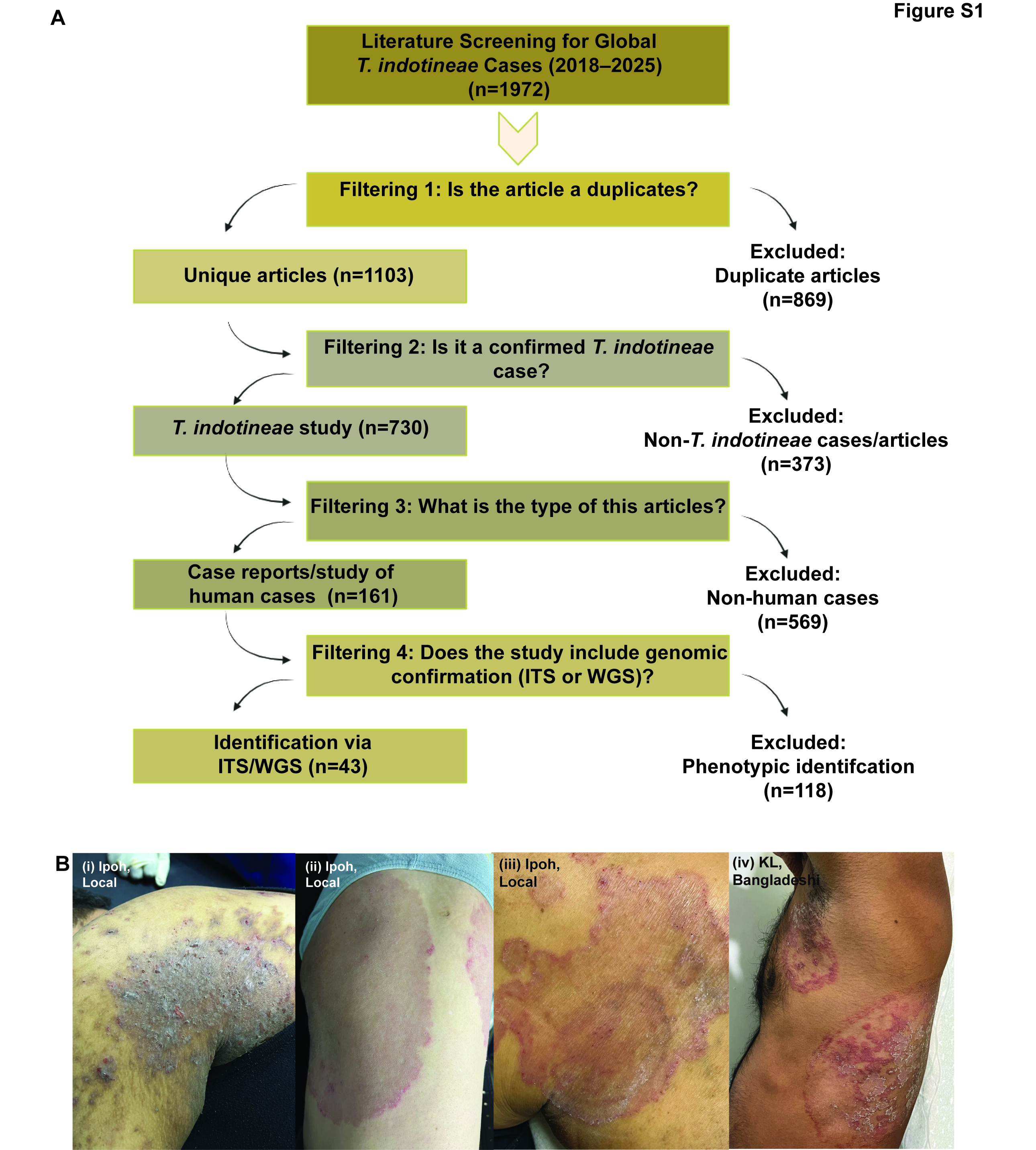

Supplement: Supplementary file 1 [file jof-11-00523-s001.zip › Fig_S1_0107.tif]
